# Equine pericardium: a versatile alternative reconstructive material in congenital cardiac surgery

**DOI:** 10.1186/s13019-021-01494-y

**Published:** 2021-04-23

**Authors:** Ahmed Abdelrahman Elassal, Osman Osama AL-Radi, Zaher Faisal Zaher, Ahmed Mohamed Dohain, Gaser Abdelmohsen Abdelmohsen, Ragab Sayed Mohamed, Mazin Adel Fatani, Mohamed Esam Abdelmotaleb, Nada Ahmed Noaman, Mahmoud Akl Elmeligy, Osama Saber Eldib

**Affiliations:** 1 Department of Surgery, Cardiac Surgery Unit, King Abdulaziz University, Jeddah, Saudi Arabia; 2 Cardiothoracic Surgery Department, Zagazig University, Zagazig, Egypt; 3 Department of Pediatric Cardiology, King Abdulaziz University, Jeddah, Saudi Arabia; 4 Pediatric Cardiology Division, Department of Pediatrics, Cairo University, Cairo, Egypt; 5 Department of Surgery, Umm Al-Qura University, Makkah, Saudi Arabia; 6 Pediatric Cardiac Intensive Care Unit, King Abdulaziz University, Jeddah, Saudi Arabia; 7 Department of Anesthesia and Critical Care, King Abdulaziz University, Jeddah, Saudi Arabia; 8 Department of Surgery, Cardiac Surgery Unit, Cardiopulmonary Perfusion Unit, King Abdulaziz University, Jeddah, Saudi Arabia

**Keywords:** Equine pericardium, Congenital heart diseases, Cardiac surgery

## Abstract

**Background:**

Pericardial patches are often used for repair of congenital cardiac defects. The aim of this study was to describe our initial experience with the use of equine pericardium and its safety and advantages and disadvantages compared to bovine pericardium.

**Methods:**

We designed a retrospective cohort study of 111 patients who were surgically treated for congenital heart disease between 2017 and 2020. Equine pericardium was used in 58 patients and bovine pericardium was used in 53 patients. Recorded variables included demographic data, preoperative cardiac pathology, site of patch insertion, morbidity and mortality.

**Results:**

The overall survival rate was 94.5% and no deaths were related to patch insertion. None of our patients were reoperated on for patch related complications. Postoperative transcatheter intervention was needed in 2 patients (1.8%): one for dilatation of aortic arch stenosis after repair of hypoplastic left heart syndrome with equine pericardium and one for dilatation of pulmonary artery branches after repair of tetralogy of Fallot using bovine pericardium.

**Conclusions:**

Equine pericardium is a safe patch material for reconstruction in congenital heart surgery. It may be preferable to bovine pericardium in cases requiring a complex shape or a pliable patch as in in arch reconstruction or for valve reconstruction.

## Background

Congenital cardiac surgical procedures often require patch material for reconstruction and repair. Several materials are currently used including, most commonly, autologous pericardium, bovine pericardium, synthetic polytetrafluorethylene (PTFE). Tissue pliability, competence of repair, and freedom from patch related complications are important criteria that determine choice of patch material [1]. We started to use equine pericardium as a reconstructive patch material 3 years ago. The surgeon initially used equine pericardial patch material as choice of trial and evaluation then he used it interchangeably with other patch materials specially in neonatal cardiac surgery and more complex procedures. He opined that although equine pericardial patch was soft, pliable, thin and remodeled in proper shape, it was enough tough for blood sealing and to counter dehiscence and aneurysms. We try her to provide observational study about equine pericardial patch material in pediatric cardiac surgery as we notice that subject is not widely published in literatures. In this study we present our experience with the use of equine pericardium in a wide range of congenital cardiac surgical procedures and how it compared to bovine pericardium.

## Methods

**Fig. 1:**
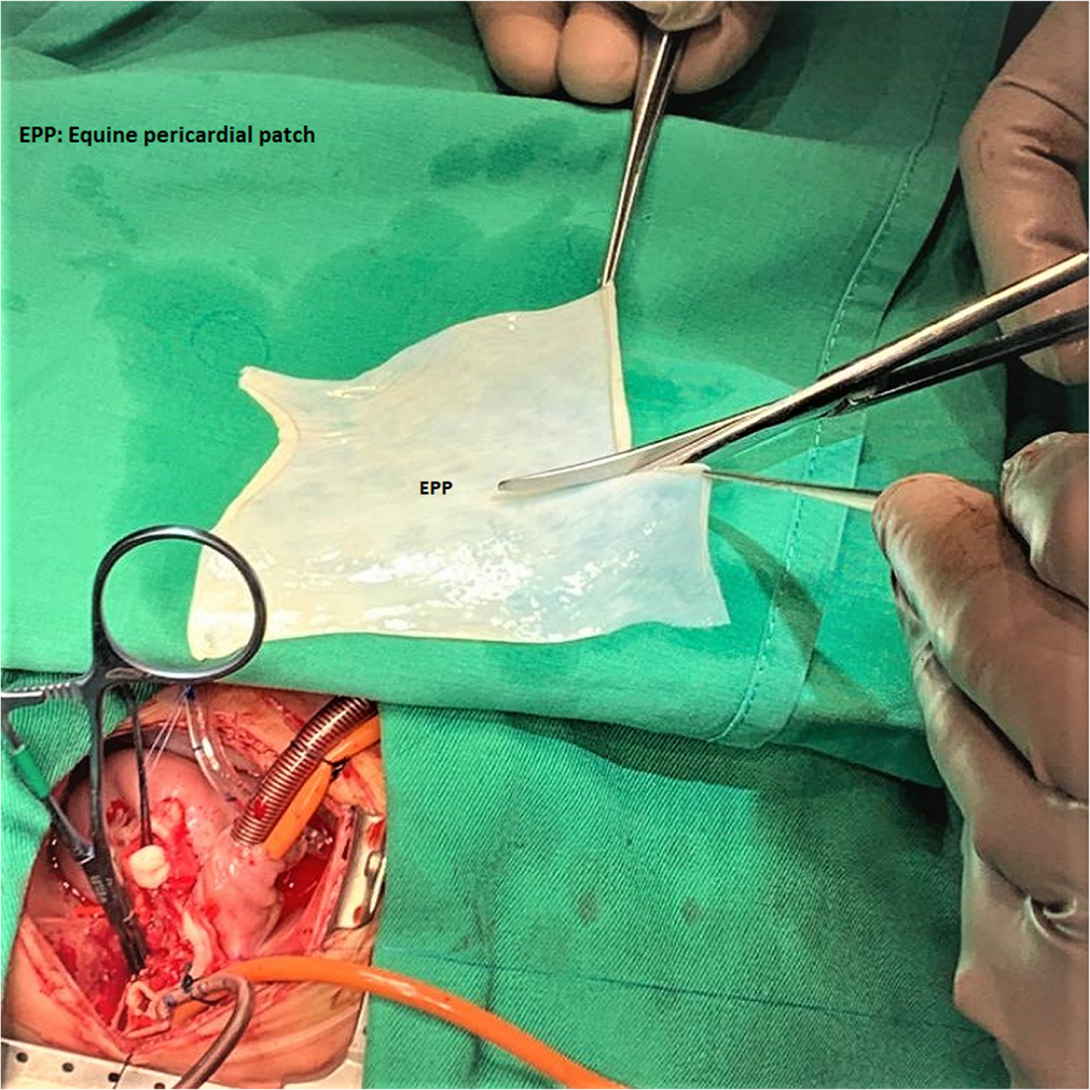
Intraoperative image during repair of distal part of ascending aorta and aortic arch by using equine pericardial patch in one-month baby presented with truncus arteriosus with interrupted aortic. EPP: Equine pericardial patch

**Fig. 2:**
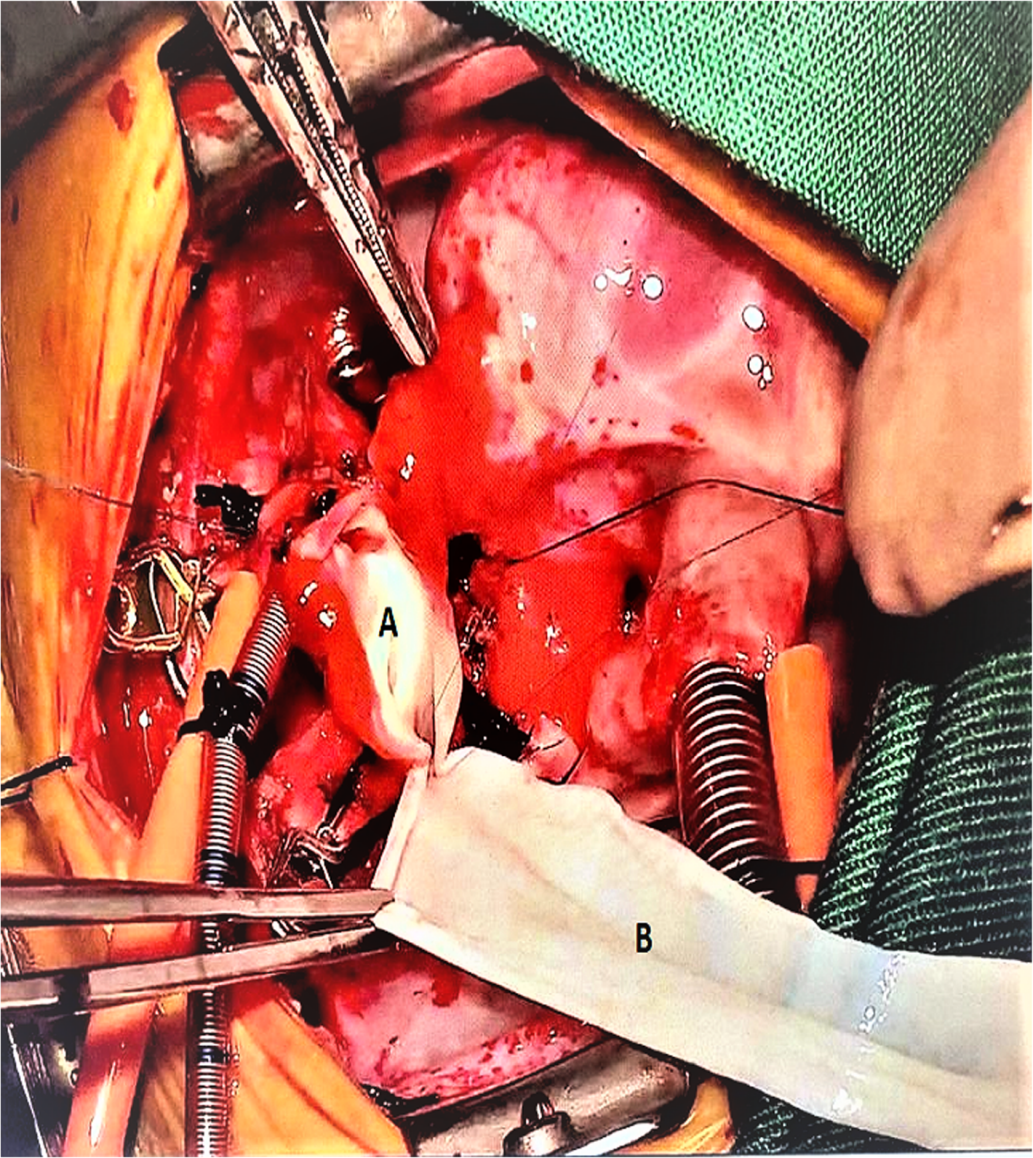
Intraoperative image of the baby in Fig. 1. **a** Distal part of the aorta and aortic arch, **b** Equine pericardial patch

This retrospective cohort study has been approved by Ethics Committee (EC) on 28/9/2020 and under reference number: 503–20. The consent of patients obtained. All patients who underwent any procedure with the use of equine pericardium at our institution were included and This included patient from 2017 till the study date. Patient and procedural data were collected in a prospective clinical database. Additional review of the medical records was also utilized to obtain information about possible complications and reinterventions. The equine pericardial patch (Matrix patch™) manufactured by Auto Tissue, Berlin GmbH and certified by TÜV Rheinland LGA Products GmbH (Fig. 1) was used. The material in approved for clinical use by the Saudi Food and Drug Authority which regulated all medical and surgical materials including implants. We included patients who underwent congenital cardiac surgery with the use of bovine pericardium (SJM™ pericardial patch) in the same time period as a control group. The choice of patch was made by the surgeon based on the type of repair. Generally, the surgeon preferred equine pericardium for repair requiring a complex shape patch, for example in arch reconstruction (Fig. 2), or in valve repair requiring tissue pliability. Bovine pericardium was used for simple patch shapes, for example right ventricular outflow augmentation. We excluded from the study the cases who were suitable for repair by cost effective autologous pericardial material like repair of secundum and primum atrial septal defects, sinus venosus atrial septal defects with partial abnormal pulmonary venous connections, repair of partial atrioventricular canal and any discrete pulmonary artery stenosis specially supravalvular one. We assessed the patients after surgery by clinical evaluation and echocardiography as a routine for all cases and cardiac catheterization for selected cases. Accordingly, we can determine any residual lesions, other morbidities and mortality. Variables collected for comparison included: age, gender, weight, body surface area, Risk Adjustment in Congenital Cardiac Surgery (RACHS) score, preoperative cardiac pathology, site of patch insertion, morbidity, reinterventions and mortality related to patch.

### Statistical analysis

Frequencies were presented as absolute number and percentage. Continuous variables were presented as median and range or mean ± SD. Pearson’s chi-squared test was used to analyze qualitative variables. Mann-Whitney rank sum test was used for continuous variables. A *p*-value < 0.05 was taken as statistically significant.

## Results

Total number of patients was 111 patients, 58 repaired with equine pericardium (EP group), and 53 repaired with bovine pericardium (BP group). Patients’ characteristics are shown in Table 1. No statistically significant difference was found between the two groups regarding age, gender, weight and RACH score. Patients in EP group had smaller BSA than those in BP group. The most common primary cardiac pathology was PA/VSD (18.9%) and HLHS (12%) in EP group and TOF (43.4%) in BP group. The most frequent site for patch insertion was main pulmonary artery (26.5%) and aortic arch (26.5%) in EP group and RVOT (45.2%) and VSD (30%) in BP group (Table 2). Follow up was available for 100 patients (95.2% of survivors). Mean follow up period was 1 ± 0.67 year. Outcome of patch repair is shown in Table 3. Residual stenosis and shunt were insignificant and did not necessitate intervention in both groups apart from 2 patients. The first was in BP group where transcatheter dilatation of right and left pulmonary arteries was done after repair of TOF during the same admission. The second patient was operated for HLHS using EP. Postoperative echocardiography showed high gradient along the aortic arch and was dilated by balloon dilation with good response. No other patch related complications (e.g. infection, dehiscence, or bleeding from suture lines) were found in either group. No mortality was related to patch insertion.

**Table 1:** Characteristics of patients

	EP	BP	*P* value
Number of patients	58	53	
Age (median, range) .	343.5 days (2–1439)	531 (14–9223)	0.06
Gender M/ F	32/26	34/19	0.33
Weight	10.96 ± 11.33	14.15 ± 14.46	0.08
BSA	0.46 ± 0.33	0.58 ± 0.33	0.03
RACHS	5.5 ± 2.1	5 ± 1	0.11
Primary diagnosis			
Left sided lesions			
HLHS	7	0	
Coarctation/ hypoplastic aortic arch	5	0	
Interrupted aortic arch	2	3	
Supravalvular aortic stenosis	3	0	
LVOT obstruction	0	1	
Mitral stenosis	1	1	
Mitral regurgitation	2	0	
Congenital aortic stenosis	1	1	
Aortic regurgitation	3	0	
Right sided lesions			
PA/VSD	11	4	
DORV	3	3	
RVOT obstruction	1	0	
Pulmonary artery stenosis	2	1	
Pulmonary valve stenosis	0	1	
Other			
Truncus arteriosus	1	2	
TGA	5	3	
TOF	7	23	
VSD	2	5	
AVSD	0	4	
Aortopulmonary window	0	1	
Endocarditis	1	0	
Pulmonary vein stenosis	1	0

**Table 2:** Sites of patch insertion

	No. of EP patches	No. of BP patches
RVOT		
Infundibular	10	15
Transannular	0	9
Pulmonary arteries		
Main	16	10
Right	7	0
left	8	1
Aorta		
Root	5	0
Ascending	9	2
Arch	16	1
Descending	1	2
Defects		
VSD	0	17
Valves		
Aortic	4	1
Mitral	3	1
Tricuspid	1	0
Pulmonary	1	1
Others		
Innominate vein	1	0
Pulmonary veins	1	0
LVOT	0	1
Total no. of patches	83	61

**Table 3:** Outcomes of patch repair

		Number of cases with EP	Number of cases with BP	*P* value
Recurrent stenosis				
Main pulmonary artery		3	2	
Pulmonary artery branches		0	1	
Aorta		2	0	0.26
Pulmonary veins		1	0	
Residual VSD		0	1	
Need for catheter intervention		1	1	
Mortality	Total No.	5 (8.6%)	1 (1.88%)	0.11
	Causes of death	Intracranial hemorrhage (1)	RV failure (1)	
		Pulmonary hemorrhage (1)		
		Respiratory failure (2)		
		Low cardiac output (1)		

### Limitation of the study

This is study is retrospective single center experience study of small volume and follow up is quite short. Prospective study of large volume with intermediate and long term follow up will provide a more evidence-based conclusion.

## Discussion

Surgical procedures for correction of congenital cardiac diseases are often accomplished with use of patch material. Autologous pericardium is not usually sufficient especially in reoperations or staged procedures. A diverse range of patches including synthetic or xenogeneic are available as tissue substitutes. Ideal patch should be readily available, easy to handle, has growth potential, can recellularize, remodel, resist infection and coapt well to suture lines for proper hemostasis and lower thrombogenicity [2, 3]. Unfortunately, none of the available patches meet all of these criteria. Synthetic patches e.g. Dacron and Gore-Tex cannot remodel, regenerate, nor grow. They are liable to infection stiffening and calcification over time after implantation [4]. Bioprosthetic patches show better surgical handling and more resistance to infection than synthetic patches. Processing of xenopericardium (decellularization) is important to remove cellular antigens and procalcific materials while maintaining integrity of extracellular matrix. In addition, cross linking (by agents e.g. glutaraldehyde) increases stability and strength of tissue and keeps it non-antigenic [5, 6]. However, late calcification is not uncommon and may be related to the type of processing and decellularization as well as the anticalcific treatment.

Tissue engineering is adopted to avoid some of these drawbacks. The bovine pericardial patch (CardioCel) is treated to remove antigens and calcium binding phospholipid sites, thus limiting calcification and reducing reoperation [7]. Dye-mediated photo oxidation is an alternative to glutaraldehyde for cross linking of collagen fibers in bovine pericardium (Photofix) [8].

Limitations related to availability and costs of these types are considered in our choice. We are still using autologous and bovine pericardia for repair. Equine pericardium has been introduced at our center for the last 3 years. EP is a decellularized patch not fixed with glutaraldehyde. Subjectively we found it softer, more pliable, and easier for handling especially in areas requiring a complex patch shape. It shows excellent adaptation to tissues notably in reconstruction of the aorta in neonates with HLHS or other complex arch pathology.

An interesting animal study by Dohmen et al. showed favorable characteristics of EP. Decellularized equine pericardia were implanted into the descending aorta of juvenile sheep. Explanation was done after 4 months. There was no evidence of thrombosis, infection, calcification, or degeneration. Extracellular matrix was preserved. A monolayer of endothelial cells was noticed on the inner side of the patch and neovascularization was found in the outer side. This study showed remodeling and regeneration of equine pericardium [9].

EP is successfully used in varying surgical sites. EP is also used as dural substitute. It is transparent, impermeable to CSF, does not adhere to cortex and facilitates regeneration of dura. It has the advantages of greater physical resistance and less liability to infection than bovine pericardium [10]. EP was also used in myringoplasty (to close tympanic membrane perforation). Long-term closure rate was better in EP group compared to BP (bovine pericardium). EP is thinner, easier to handle and remodel in proper shape than BP [11]. EP is used in treatment of chronic wounds and ulcers of diabetic foot. It provides temporary biological cover scaffold that promote healing [12]. Few data are found in literature regarding use of equine pericardium in cardiovascular surgery. EP was approved for pediatric cardiac reconstructive surgery and was successfully used as a substitute to arterial homograft to replace infected aortic aneurysm [13, 14]. Equine pericardial patch is used to close the pericardial sac to decrease risk of repeat sternotomy. Lesser adhesions were found in equine pericardial patched group when compared with pericardium left open group [15]. On the contrary others reported intense epicardial reactions, degeneration, and calcification. This discouraged most surgeons from using xenopericardium for closure of pericardial sac [16]. We do not routinely close the pericardium in pediatric patients. We think that glutaraldehyde added to fix pericardial patch might account for these undesirable changes. Accordingly, glutaraldehyde-free Matrix patch ay be a better alternative. An experimental study was conducted by Rassoli et al. compared equine, bovine, and porcine pericardia mechanically and histologically. Equine pericardium showed less stiffness under biaxial tension and hence it is more appropriate for manufacturing bioprosthetic valves as recommended by authors [17]. EP was used to construct a stentless bioprosthetic valve with good hemodynamic results comparable to Toronto SPV valve as shown in an animal study by Muller and Segsser [18]. We used EP for augmentation of valve repair in 9 cases with good results in terms of coaptation and competence. We prefer to do bicuspidization of pulmonary valves with EP. Early follow up results of our series showed favorable outcomes of EP comparable to BP with respect to survival and freedom from reoperations. A few clinical trials compared the use of EP and BP in surgery for congenital cardiac diseases. Vitanova et al. reported higher rate of recoarctation after Norwood stage 1 for HLHS when equine pericardium was used for reconstruction of aortic arch in comparison to homograft, autologous pericardium, and bovine pericardium. They concluded that equine pericardium was the only risk factor for recoarctation and cannot be recommended for repair of HLHS [19]. We operated 7 patients in our series with HLHS using EP. Transcatheter dilatation was needed for one patient and responded well to dilatation. The remaining patients had excellent arch reconstruction using EP and many have undergone second and final stage surgery without evidence of significant calcification or stenosis.

The cost of different materials varies in different countries. However, in Saudi Arabia the coast of the EP is comparable to BP (EP cost is 900 dollars and BP cost is 825 dollars). We agree with Veličković et al. [20] that the rate of revision or reoperation related to patch failure should be considered when economic aspect is evaluated.

This study is a retrospective single center study including a relatively small number of patients with different congenital cardiac diseases. Prospective studies with larger sample size and longer follow up period are needed to evaluate long term outcome.

## Conclusions

We conclude that equine pericardium is safe and efficient tissue substitute for repair of congenital cardiac defect with comparable results to bovine pericardium and is preferable in our experience for cases requiring complex arch reconstruction or valve repair.

## Data Availability

The datasets used and/or analysed during the current study are available from the corresponding author on reasonable request.

## Abbreviations

PTFE: Polytetrafluorethylene
RACHS: Risk Adjustment in Congenital Cardiac Surgery
EP: Equine pericardium
BP: Bovine pericardium
BSA: Body surface area
PA/VSD: Pulmonary atresia with ventricular septal defect
HLHS: Hypoplastic left heart syndrome
TOF: Tetralogy of Fallot
RVOT: Right ventricular outflow tract
